# An empirical and philosophical exploration of clinical practice

**DOI:** 10.1186/s13010-019-0072-9

**Published:** 2019-02-21

**Authors:** Michael Saraga, Donald Boudreau, Abraham Fuks

**Affiliations:** 1 Liaison Psychiatry, Lausanne University Hospital, Av. de Beaumont 23, 1011 Lausanne, Switzerland; 2 Center for Medical Education, Faculty of Medicine, McGill University, Montreal, Quebec Canada; 3 Department of Medicine, McGill University, Montreal, Quebec Canada

**Keywords:** Clinical practice, Patient experience, Hermeneutic phenomenology, Qualitative research

## Abstract

**Background:**

Previous empirical work among physicians has led us to propose that clinical practice is experienced by clinicians as an engagement-in-the-clinical-situation. In this study, we pursue our exploration of clinical practice ‘on its own terms’ by turning to the experience of patients.

**Methods:**

Phenomenological analysis of in-depth individual interviews with 8 patients.

**Results:**

We describe the patient experience as a set of three motifs: the shock on the realization of the illness, the chaos of the health care environment, and the anchor point provided by an engaged physician. We draw on Heidegger’s notion of solicitude to show that patients are actively ascertaining the physician’s engagement in their care.

**Conclusions:**

These findings lead us to question the classical “dual discourse” of medicine that offers a dichotomous account of clinical practice as the addition of care to cure, art to science, humanism to technique, and person to medical case. We found no such distinctions in our empirical investigation of clinical practice. Rather, in our synthesis, practice appears as a unitary experience. The physician’s solicitude for the patient entrains engagement in the clinical situation. Moreover, the solicitous, engaged physician constitutes an anchor point for the patient.

*[…] we do not just want to know what virtue is, but to know it in order to become good.*

Hans-Georg Gadamer, *On the Possibility of a Philosophical Ethics* ([1], p. 277)

## Background

Common, though often implicit, understandings of clinical practice frame it as applied medical science. In such a view, the practitioner applies scientific knowledge of the bodily processes of diseases and implements the specific strategies that have been demonstrated to be appropriate, usually on a base of statistical studies on groups of individuals. In a sense, this can be considered an idealistic approach to clinical medicine: truth is to be found outside of practice itself, in the realm of abstract, universal, scientific ideas. Moreover, if one defines clinical medicine as an applied science, the question arises as to how physicians should account for the humanity of their patients. The “science” of medicine is then seen to require a complementary “art” [2]. Such a dual account of practice implies a sharp distinction between the domains of science (objective, rational, universal, value-free) and of the human (subjective, emotional, relative, value-laden). This implicit understanding of clinical medicine has been described as a “dual discourse” by medical anthropologists [3].

As such, the dual discourse has not been the object of much criticism. It appears as a self-evident truth: medicine as the addition of the science of the body and the care of the person. Reasons to doubt the validity of the dual discourse as an account of clinical practice can be found outside of medicine. Notably, phenomenology and anthropology have developed views of practice as embodied, situated, and only partially conscious. This invites practice to be understood “on its own terms” and not as the application of theoretical rules [4].

As physicians, we find merit in such criticisms, and the dual discourse appears to us a poor account of clinical practice. This was our motivation in initiating a broad research program to study the nature of clinical practice. Our initial step was an examination of clinical medicine as an epistemic practice, using George Engel’s biopsychosocial model as a lens [5]. Engel noted that what he called “biomedicine” can be applied only to the body. Yet, the actual epistemic object of clinical practice is the patient; it therefore calls for a biopsychosocial paradigm [6]. However, we noted two limitations of Engel’s model. First, it is highly abstract and does not account for how a seasoned physician actually thinks, feels and acts in practice. Second, for Engel, the patient is the focal point of his model. That may result in an underappreciation of the physician’s biopsychosocial situation, namely the context of clinical practice.

In response to these limitations, we decided to study, empirically, clinical practice as a lived experience. In a first study [7], we explored the lived experience of the physician at work: specifically, how physicians think, feel and act in a contextualized practice. The object under examination was therefore clinical practice, defined as the experience of the physician at work. We used a phenomenological approach and worked with clinicians who had been selected by colleagues as ‘exemplary’ physicians. Our analysis led us to propose that clinical practice is best understood as an “engagement-in-the-clinical-situation.” Engagement provides an account of clinical practice as a unitary lived experience. Drawing on Aristotle’s notion of *phronēsis* and Sartre’s definition of the situation, we illustrated how this novel perspective captures the physician in the clinical situation, including a context of materialities, procedures and colleagues.

In this article, we present the results of a complementary empirical study. We explored the same object, namely clinical practice, this time through the *patient*’s lived experience of the physician at work. It seemed relevant to include in our investigation the patient’s experience, as the patient is arguably the object of clinical practice and, at the very least, a privileged witness. We will proceed by first describing, in detail, this second study, and then discussing how the findings of both studies are related.

## Methods

Our study is based on interviews with 8 “patients-as-partners” affiliated with the Université de Montréal. The concept of patient partnership is relatively new and warrants a description. A patient-as-partner is a person suffering from a disease, usually severe and chronic, who is actively engaged in his or her own care, but also, more broadly, in institutional projects within academic and/or healthcare organizations, in relation to teaching, research, or various procedures [8]. More rarely, a patient-as-partner is a close relative of the actual patient. For our study on the physician’s experience, we had chosen ‘expert physicians,’ recognized by their peers for clinical excellence, experience, and capacity to reflect on their own practice. Analogously, we chose to work with patients-as-partners, who are, in a sense, ‘expert patients.’ Patients-as-partners are, or have been, confronted with serious diseases. They have an extensive experience of healthcare, and have had numerous different encounters with clinical practice. Positioning themselves as partners of medical institutions, they are attentive witnesses of medicine, engaged in a reflective process on the nature of health care, and thus able to provide rich accounts of their experience.

The participants were identified, approached by the appropriate office at the Université de Montréal and provided informed consent. They received a modest compensation for their time. The final sample included 3 women and 5 men, aged between 24 and 70 (see Table 1).

**Table 1:** Participants

Virginia	Locally invasive tumor of the orbital floor
Nathalie	Systemic lupus erythematosus
Jacques	Chronic lymphoma
Josué	Crohn’s disease
Antoine	Cystic fibrosis
Esther	Breast cancer; husband with Alzheimer’s disease
Patrick	Son with congenital renal insufficiency
Pierre	Type 1 diabetes with multiple complications

We conducted in-depth interviews (carried out by the first author, a psychiatrist with 15 years of clinical experience, who was at the time a visiting professor at McGill University). The first meetings explored in detail their experiences of clinical practice, usually focusing on a number of specific situations that carried particular meanings for the participants. The follow-up interviews further explored relevant issues and gathered reflections and comments on the first interviews. The duration of the meetings varied between 60′ and 105′ for the initial interview and between 45′ and 75′ for the follow-up. The interview process was exploratory in nature. In particular, the interviewer pointed out contradictions when they appeared, inviting participants to clarify and further elaborate on their experience. We underline that the participants were evoking past events as they remembered them during the interview. We considered that the manner in which they described the events was indicative of their experience of the events at the time. Moreover, they were reflecting, with the interviewer, on their experiences, adding to the richness and the complexity of the material.

For the analysis, we adopted a qualitative descriptive approach with a “phenomenological overtone,” as outlined by Sandelowski [9]. Working on the participants’ narratives, we aimed to capture their experiences of the physician at work. We proceeded inductively, identifying segments of the material that constituted units of meaning, which led us, through repeated discussions, to a consensus on three synthetic motifs. They provided a reasonable and structured account of the whole material with regard to our object of inquiry.^1^

The study protocol was approved by the institutional review board of the McGill University Faculty of Medicine.

## Results

### A tale of stormy seas and safe anchors

In this section we make the case that patients experience clinical practice as an *anchor point* offered by an engaged physician. The anchor point takes its meaning against the backdrop of the *shock* of the illness and the *chaos* of the health care environment. We present these findings in a sequence, a meta-narrative which helps to underline how they interact dynamically. We stress that all the individual interviews did not necessarily follow this precise structure. Nonetheless, the three motifs link sequentially, providing a faithful integrated account of the individual narratives. The coherence between this account, comprising the three identified motifs, and the participants’ individual stories, argues for the findings’ relevance.

We illustrate our findings using direct quotes from the interviews.^2^

### The shock

In the participants’ narratives, there was often an initial shock. The shock can affect the body—the experience of a painful, failing, unresponsive body. For instance, for Nathalie:

During the Christmas period I was in an extreme fever, I had a loss of weight, I wasn’t eating anymore, really an extreme weight loss, I had pains, I would wake up perspiring, my mother had to put ice on me. I wasn’t living with my parents but I spent the entire holiday period at my parents, my mother would put cream on me because I was hurting everywhere and then in January I began my PhD and I was happy. I had no more pain anywhere, and one week later I was… I was falling asleep while the teachers were reading the syllabus. I was unable to keep awake, unable to move and so one weekend my mother told me: “Nathalie, what are you doing? Are you spending Monday on the sofa, what are you doing? Will you go to your lecture or will you stay forever on the sofa?” And so on Sunday night I went to the hospital, I arrived in extreme fatigue with a body hurting to the highest degree, I was unable to move when I went to the hospital. I couldn’t get out of bed, go to the bathroom, I was… my hands were so cramped, if you want, that I couldn’t wipe myself, I was in a state, I wouldn’t say of degradation but almost.

The shock can be caused by a single word: “tumor,” “cancer,” “surgery”—an announcement that tears apart the existential fabric. For Virginia, it is a benign tumor of a facial bone, locally invasive and quite large. The shock comes from her otolaryngologist, who is, in her words, “so kind, so warm, so reassuring, so sensitive, whose gaze is so trustworthy.” Yet, as he describes the surgery and the risk to the optic nerve, the shock is her sudden, overwhelming thought: “Oh my God, I will lose my eye, this is over.”

Sometimes the shock is less overt, perhaps a story hidden within another story. Antoine, who suffers from end-stage respiratory failure, learns that he needs not only a lung transplant, but a double, lung-liver transplant, the first to be performed in the region. When he visits the liver transplant clinic, he is “stunned” that the young resident does not seem to know anything about his complex medical situation. This “stunning” encounter can also be understood as an echo of the previous shock of learning about the necessity of a double transplant. Jacques, who has been diagnosed with a lymphoma, is “shocked” that the oncologist does not invite his wife to participate in the consultation. But, quite likely, the shock is also that he, a senior administrator of that very same hospital, must now become a patient and finds himself forced to entrust his care to this intimidating physician.

We do not claim that patients always experience illness as a dramatic “shock.” An apparently minor disruption can still be significant. For example, learning that one must take an anti-hypertensive drug daily, perhaps forever, may seem routine to a physician, yet can be upsetting to a patient by signaling the limits and ineluctable decline of the body.

The shock is a catastrophe, current or impending, that strikes the participants’ most intimate selves. Edmund Pellegrino [10] described disease as an “assault on the ontological unity of body and self”, underlining that it strikes at the very being of the person (p. 44). Kurt Goldstein [11] spoke of a “shock and danger for existence” (p. 329), manifest in “catastrophic reactions” to the ordinary milieu, in which the patient is “buffeted, and vacillating [, experiencing] a shock affecting not only his own person, but the surrounding world as well” (p. 49). This very real and material danger is the continuing backdrop against which the whole clinical experience unfolds. Indeed, the shock is also the trigger of subsequent events. Existence seems to fold and redeploy in a new, very different way. Actions must be taken; help must be sought. The person transforms into a patient and enters the “health care system” [12]. Thus begins the second stage of the tale.

### Chaos

In the words of Nathalie, entering health care is venturing into a chaotic world:^3^

It’s chaotic. When students ask me, when they pose the question: “But how is it? Really?”—this is chaos, you’re entering into chaos.

The participants experience the health care environment as unresponsive and absurd, to a degree reminiscent of the worlds of Franz Kafka and Lewis Carroll. Examples in our material abound; we provide one from Josué:

I would call; that was a rotating personnel so it wasn’t always the same person who would set the appointments and they had no clue about my name, or anything […] when I called my surgeon it was voicemail upon voicemail and then, I talk to I don’t really know who, who’s in an office I don’t know really where; and they, I don’t know if they are able to see the surgeon, if they’re able to transmit the message… I guess it’s only an appointments-desk. So, if I have a problem, there’s no one in that hospital for me.

[…] I got myself there and they didn’t even know why I was there […] I got myself there two or three times for nothing. They would look at me, and tell me: “I don’t know why you’re here, I can’t examine you.” […] I had a surgeon who was examining me for an open wound that should heal. And at a certain moment when the wound was stagnating, and he couldn’t close it, he told me: “I’ll have you meet a plastic surgeon who will fill it up.” When I came to see the plastic surgeon, he examined me and told me: “I don’t understand what the surgeon wants me to do. I don’t know what to do with your case, I’ll talk to him and you’ll come back in three weeks.” When I returned three weeks later, he hadn’t talked to the surgeon, he didn’t have any more ideas on my case.

In the chaos, people cannot be reached, or, when they are, they cannot answer the questions asked. When unforeseen events happen, they are minimized or even denied, as when Virginia wakes up after her surgery to find out that the surgeon has used fatty tissue to fill in a deficit in her left orbital floor:

When I woke up, in my hospital bed, there were four friends of mine and my parents. And they were staring at me, all six, with… you know… looking all traumatized. I couldn’t understand why, and then, I don’t remember who… one of the six said: “Don’t panic, you know, but you have [laughs] a hole in your head.”

Subsequently, furious at what had happened, and especially that she had not been forewarned that it might happen, she quarrels with the resident. Later that same day, when the surgeon comes to see her, he insists that he had warned her.

#### Dangerous decisions

However, this chaotic world is not only difficult to navigate, unresponsive and apparently absurd—it is fraught with the danger of mistaken clinical interventions. Decisions are made and actions taken, seemingly coming from nowhere, for no apparent reason. For instance, one Friday night, two residents announce to Nathalie that mycophenolate, her treatment since the diagnosis of a severe auto-immune condition, will be stopped and replaced by cyclophosphamide. The decision had been made by the chief immunologist on the ward without any discussion with her, he was not there to discuss it now, and the residents were unable to explain the rationale for the change. For Nathalie, this is an example of a physician who does not engage in what she calls an “egalitarian relationship,” a “partnership” with the patient: the immunologist is taking the decision all by himself, without any discussion with her. However, the problem here is not only one of discussing and sharing the clinical decision or, more broadly, proper “bedside manner” [13]. The problem, for Nathalie, is above all that cyclophosphamide is toxic, and might have negative repercussions on her fertility, an issue she had discussed at length with her nephrologist. They had agreed that it should remain an option of last resort. Two things, therefore, need to be distinguished. First, is an issue, almost political, of power balance in the clinic, in that the immunologist takes a decision “about her without her” [14]. Second, and most importantly for Nathalie, is that the immunologist makes a decision that might well be a bad decision for her, a decision putting her in danger. Indeed, in Nathalie’s narrative, it was clearly a poor decision, as she refused the therapeutic change, remained on mycophenolate, and improved.

Chaos is not limited to unfortunate problems of coordination, communication or management, the result of organizational difficulties among well-intentioned professionals. Physicians themselves contribute to this sense of chaos, insofar as their decisions appear at times nonchalant, incomprehensible, and possibly dangerous. What is most important is that the chaotic situation is one of danger, a very material danger for the patient. In contrast to Carroll’s seemingly invulnerable Alice, patients are indeed extremely vulnerable. Our participants’ diseases were serious, requiring demanding treatments. The clinical decision, this surgery, that prescription, a transfer to another hospital or clinic, the decision to be placed on the organ recipients list, the errors, the conflicts, the misunderstandings—these can all have grave clinical consequences.

#### Walking on eggshells

The situation is made worse by the fear that the physician might get upset if the patient protests or asks for explanations. The physician is making decisions that might be dangerous, but there is also danger in questioning them. Participants often described how they attempted to appear as “good patients,” lest they be perceived as “bitchy patients,” as Nathalie said. This is the danger for Patrick, whose 3 year-old son has been on dialysis for a mysterious disease since birth. The boy had already undergone more than 30 surgeries; the dialyses were becoming more and more difficult and his life was at stake. To say the least, it was a very dramatic situation. Patrick wishes to seek a second opinion on the advisability of a renal transplant:

Patrick: […] at that moment I suggested to the doctor, “Is it be possible to check with Hospital X, to have them, without necessarily going to Hospital X, but go ask them, send them the file and see what they think and what they would suggest?” and on and on and on. And this physician was, I was a bit afraid to talk to her about that, I didn’t want to offend her.

Interviewer: Why were you afraid of this?

Patrick: Because in the relationship I still have with the physicians, it is somewhat of a relationship where they have the last word on my son’s condition, they are the ones keeping him alive, you understand. It’s we who do the work around this but at some point my son’s life is in their hands. Me, what I really didn’t want was to antagonize a physician. I didn’t want to get to the point where they don’t like my face; that would make things more difficult, and then I would tend to seriously question what they do or don’t do, whether they do what they should, because there would be this tension of whether they do… well—I wanted to avoid this at all cost. […]

Interviewer : You were anxious…

Patrick: Very anxious, I discussed this with my partner, and then we came to the conclusion that we had no choice… look, it’s my son’s life, he will come first.

[…] these are difficult decisions, that keep us awake at night, and we don’t always agree, my partner and I […] before going to see… we discussed a lot […] we both had the same fear [of the doctor’s reaction].

These parents, who were going through such an ordeal with their son, were agonizing and arguing at night about whether it might be dangerous to ask the physician for a second opinion. In this case, the fear was indeed misplaced, as the physician responded with kindness (and tears, as we will discuss below) to their request. Nevertheless, it illustrates how intensely patients sometimes fear the reactions of health care professionals. “Walking on eggshells” is an exercise in caution, the painful necessity that comes with disease, illustrating how dealing with a chaotic health care environment and unreliable health care professionals can heighten the distress caused by illness and its consequences. Many health care professionals probably do not appreciate, let alone acknowledge, the specific distress they can induce in patients. Indeed, physicians have a tendency to take for granted that the patients will trust them, and to posit trust as a *condition* of clinical care [15]. In our material, trust was rather a *result*, the result of finding a physician providing an *anchor point* in the existential and situational tempest that the patient was facing. This is our third finding, discussed below, that points to the proper nature of clinical practice.

### The anchor point

#### Finding connection and the medical “chalet”

In some interviews, participants described specific moments where they found a sense of connection with the physician. It could be an instant where they perceived tears in the physician’s eyes, a glimpse of the “person” behind the “professional.” It could be a way of looking at them “in the eyes.” It could be a promise to call back, and a promise kept. Nathalie tells of a very evocative instance of such connection:

At that moment this fellow enters the hospital room and tells me, “well now, we’ll meet in two weeks, the biopsy should arrive”—because I had had a biopsy, so “The biopsy should arrive within ten days, but we’ll meet in two weeks, in two weeks, we’ll review the results.” And then I look at him and say, “Ah, it’s you the physician we were waiting for.” And he said yes. “Is it you who will be my physician?” I’m very naïve, I’m excessively ill, “will you be my physician?” […] I ask him, “Is it you I will see outside?” and he said yes. I say, “Then you’re the one who’s stuck with me?”, and he looks at me and he says, “No, it’s you the one who’s stuck with me”, and there was this little moment when we both laughed; he signed a paper and he went away.

Thereafter, for Nathalie, the hospital will lose some of that chaotic quality:

My three first years, the hospital was my chalet—I went to the hospital for respite. It was a safe place for me despite the infections and the missed medication.

If the hospital, previously chaotic, is now a safe “chalet,” it is because of that same physician. Indeed, the safety of the “chalet” remains fragile, at risk of going ‘helter-skelter:’

I said to my father, “Dad, go see his secretary on the 2^nd^ floor and let him know that I am currently admitted” […] I needed to be sure that he knew I was there, I needed to be sure that it wouldn’t go haywire.

It was indeed Nathalie who spoke of an “anchor point”:

I remember that he was really my anchor point, it was he, the person I trusted—that I trusted most in the world at that moment. When I talked to him, I had such a trust in that man, for everything.

All participants described a specific physician, and sometime more than one, as providing such an anchor point. They evoked these experiences with words such as “humanity” and “partnership.” Nathalie described what constitutes, for her, a “good physician,” insisting on humanity and respect, and what she called an “egalitarian relationship.” In a slightly different tone, Virginia underlined her otolaryngologist’s “empathy.” In the interview, she described him as “reassuring.” However, he has just announced that she will need surgery and she is thinking, “My God, I will lose my eye,” which is not exactly a reassuring thought. The interviewer confronts her with the contradiction:

Interviewer: Yet you were rather very worried?

Virginia: Yes, yes, I was very worried.

Interviewer: What did you mean then, when you said, “reassuring?”

Virginia: You know, at that moment, that was not a moment when I was reassured. It was rather a moment… it’s more the way it was transmitted, you know. It’s just the fact of demonstrating sensibility, warmth, empathy. I find it played a big role you know. Because, when I think back, in retrospect, yes, it was very anxiogenic for me, I had a crisis in his office and all. It was really, it was a shock. At least, at least I felt he had empathy for me. I didn’t feel… you know, I felt like a human being… not like… like in plastic surgery, like a puppet.

Virginia is insisting on the physician’s warmth, empathy, or perhaps simple kindness. Similar notions were present in many interviews: the physician as an anchor point was described as treating patients as human beings and not objects, respecting their desires and priorities, informing them transparently, and establishing something of a closer, warmer, less strictly “professional” relationship with them. At this point, it may seem that our material faithfully echoes what has been described as the “dual discourse” of contemporary medicine: the idea that patients need not only to be ‘cured,’ but also ‘cared for,’ that empathy is needed in addition to technoscientific competence.

However, we will argue that this is a somewhat superficial understanding of our participants’ narratives. They did not describe experiences of good clinical practice as ones in which ‘care’ was added to ‘cure.’ Rather, the issue is whether the physician is really ‘doing the job.’

#### Doing the job

We have now reached a crucial, and perhaps complicated point in our argument. It is commonly accepted that empathy, warmth, respect, a humane attitude, are good things in themselves, expected to somehow help, and “reassure” the patient. Indeed, such received wisdom is also present in our participants’ narratives. However, as we delved more deeply in our participants’ descriptions during the interviews, and revisited these issues in our analyses, we arrived at a rather different understanding.

Let us begin with the idea of the “reassuring” physician. For instance, taking seriously Virginia’s claim that her otolaryngologist was reassuring to her, even though he was announcing that she might lose her eye, one is left wondering—how so? How might a response of “warmth and empathy” to Virginia’s terror be reassuring? It does not make sense if the fear that is assuaged is that of losing an eye. After all, “warmth and empathy” is not a promise that her eye will be spared. The fear that the physician seems to be able to allay, at least to some extent, cannot be the loss of her eye. Asked to elaborate further on her otolaryngologist, whom she contrasts with the plastic surgeon who would visit her only once or twice during a 2-week hospitalization, Virginia notes:

You know, just by demonstrating that *he worries about his patients* […] asking how you’re doing, first, but looking in the eyes and really showing that *he’s worried and that it’s important that I get better* and all.

In a similar vein, Jacques also states during the interview that it makes him “feel secure” that his physician, “beyond the scientific,” is also interested in him “as a person.” The interviewer invites him to explain further: how would an interest in him “as a person” make him “feel secure?”

When I see the *effort that is being made* to establish this interpersonal relationship […] The big difference is that by explaining what was going on… well… “here’s what will happen, here are the possible reactions, *I have studied your case well*.”

Jacques tells us that he finds it “extraordinary” that such a specialist has taken a whole hour to discuss the diagnosis and therapy with his family. When he explores with us why it should be reassuring that there is an interest in him as a person, he mentions the physician’s “efforts” and the fact that he has “studied his case well.”

Patrick does not talk specifically of fear, but describes his intense relief when he sees the tears in the eyes of his son’s nephrologist, as she cries when they request a second opinion, the very issue he had been discussing so intensely with his partner. The interviewer invites him to explore that memory, asking why the tears had been so important:

At one moment I remember, she was sitting at the end of the table with us and she started to cry, you know, with my wife and all, and I perceived that she, over there, she really cares […] it wasn’t going well for my son and she had accompanied us through all the steps at Hospital X, and all the discussions we had at that moment, the fact that she helped us with all the documentation, she explained to us… we spent quite some time, which we hadn’t done beforehand. *It showed how dear my son was to her*. Throughout the discussions, at a certain point, I don’t remember what we were saying at that moment, but in essence it was like, “well, there’s not much more to do for my son and we’re going to let him go.” We were talking about all that and she started to cry. I saw—you know I had always thought she was detached and all; and I could see that it was really affecting her.

Patrick is moved by the nephrologist’s tears because they are a testimony, for him, of the *importance* his son really has for her. The time she took to prepare the file and to accompany the parents in their quest for a second opinion is a further proof that she cares deeply about his son (in French: “elle a réellement mon fils *à coeur*”).

It seems that the worry shared by Virginia, Jacques and Patrick, is that the physician might not really care about the patient, might not do his best to cure the patient. This is how physicians are reassuring: when patients sense that they really care about them. In the participants’ description, the physician experienced as an anchor point is someone to whom they are important, who worries about them, who cries at the idea of bad things happening to them, who roots for them. It is not a complete reassurance; patients are still worried about the outcome of the treatment, the disease, etc. But one specific fear is allayed, that of being treated by physicians who are not really doing their best, not really doing their job.

We also wish to underline that Jacques wants his physician to study his *case* well. In the dual discourse, the patient “as a person” is often opposed to the patient “as a case”—as if a physician who sees the patient “as a case” cannot see the patient “as a person.” But Jacques is actually reassured that his oncologist sees him “as person, beyond the scientific”, because it means that he will “have studied his case well.” Being properly treated as the physician’s case is rooted in being seen by that physician as a person. In that perspective, a human relationship, in which the patient is considered as a person, is a means to the broader end of receiving proper care by securing the physician’s commitment. In other words, in the experience of the anchor point, the “case” and the “person” are like two faces of the same coin.

In sharp contrast with the negligent and prickly physician of the chaotic environment, who appears so *detached* from the clinical situation, our participants find an *anchor point* in a physician for whom it is important that they get better, who will see them as *his* or *her* “case.” Being a case, not the only case, but a unique case, for one physician, who is not the only physician, but a unique physician: that is to say, being taken care of, being the object of the physician’s dedication, diligence, and best efforts. As they reflect on their relationships with physicians, patients search for signs of such a commitment. For Nathalie, it is manifest in the physician’s strangely symmetrical response: “No, it’s you the one who’s stuck with me.” For Patrick, it is evident in the nephrologist’s tears. For Jacques, it is the time taken to meet with his family. For Josué, it is the reliability of his specialist who follows up on things.

#### A counter-example

We provide here one final excerpt that is a negative illustration of the anchor point. It clarifies what is at stake here: not only the quality of the relationship but, more broadly, adequate care. Virginia is sharing with the interviewer her anger when she wakes up after a surgery and discovers that the surgeon has performed a graft of adipose tissue that he had not previously discussed with her; her autonomy has not been respected; she feels like an object, “a puppet” in the surgeon’s hands. In the initial description of the scene, she mentioned that the resident had tried to trivialize the consequence of the graft, telling her it would be corrected easily later on with minor surgery involving a liposuction. The interviewer reminds her of the resident:

Interviewer: […] when you wake up… your anger, I’m wondering, what is feeding your anger?

Virginia: […] because I had not been warned, and then there is the other element I’ve mentioned, because I have the impression of being just a body, deprived of rights over my body, without having been asked. It was very much this I returned to, when I saw the resident. It was always, “We didn’t talk about that, he never said he would do that.”

Interviewer: But the resident, what did he say?

Virginia: […] Yes, about the liposuction yes, and that reinforced my anger.

[…] Interviewer: that meant one more surgery.

Virginia: Yes it’s true. Yes. That was part of it, I was just telling myself, “You don’t realize how much […] how much energy and resources it takes, for me, to prepare for each operation […] You don’t realize.”

[…] Interviewer: You’re thinking, maybe they took that decision a bit lightly. For them it’s not so much.

Virginia: Yes. For them it’s not so much. That’s it.

Interviewer: For them it’s not so much, for you it is a lot. […] And you would like to be certain that, if they took that decision, they did so knowing how much it would cost you […] and the fact that he had not discussed this beforehand, that leads to this question.

Virginia: Yes. That’s exactly true.

As Virginia elaborates on her experience, it appears that the problem was not simply that she had not been forewarned, that her right to be informed and to make a decision about her treatment had not been respected. She knew that this was a complex reconstructive surgery, after a bone tumor and an infection. The surgeon could not know in advance what he was going to face during surgery. The problem, so painful and infuriating for her, was that she could not be sure the surgeon had made the best possible decision. Perhaps he did not know, or did not want to take into account, how difficult it was going to be for her to undergo another procedure. She realized that perhaps there was no other choice, and if so, she was mature enough to accept the necessity of the additional surgery, the liposuction to fill up the “hole in her head.” In all likelihood, if the resident had demonstrated that the surgical team had taken that decision after careful consideration of the consequences, acknowledging that the necessity of another surgery was bad news, instead of minimizing it, it would have been acceptable. Even if she had not been forewarned. What was unacceptable was the idea that the surgeon may have taken his decision lightly, an idea made worse by the resident’s minimization of the surgery: “for them it is not much.” For her, each operation is an ordeal. It is reminiscent of Nathalie’s immunologist prescribing the cyclophosphamide perhaps not knowing, or not wanting to take into account, her desire to have a child one day. These are physicians who appear as failing to give proper, careful consideration to the patient’s existential situation.

In summary, this is where our discussion has led us thus far: clinical practice is experienced, by the patients, as an anchor point in the chaos that follows the shock of illness. However, as we stated in the introduction, the current study is situated in a research project with a broader scope, designed to explore clinical practice. This included an empirical description of the physicians’ experience of clinical practice as *engagement-in-the-clinical-situation*. We now want to bring together the results of the two studies.

## Discussion

### Engagement and solicitude

We first refine our description of the patients’ experience by turning to Heidegger’s notion of *solicitude*,^4^ which, we believe, offers some support to, and clarification of, what is at stake for the patient in the clinical situation. Heidegger draws a number of useful distinctions: relating to objects or relating to human beings, engaging the other with “deficient” or “positive” solicitude, by “leaping-in” or “leaping-ahead.” We will discuss them as they pertain to the clinical situation, and in light of our empirical material.

In section 26 of *Being and Time* [16]*,* Heidegger contrasts our experience of things, material objects, with our experience of other human beings. Things present to us as possibilities of, or impediments to, our actions, what Heidegger calls their *handiness*. In the clinical situation, the patient may well appear to the physician as one object among others. The patient is *handy*; presenting to the physician as possibilities of and impediments to medical actions. For instance, the physician may be looking for symptoms in the anamnesis or for a handy vein for insertion of a catheter. Indeed, there may be some merit in the capacity of the physician to engage the patient as a “thing-at-hand,” relating, in Heidegger’s words, with *circumspection* and *concern*, characteristic of our dealing with “things-at-hand.”

The other human being—the other “Dasein”—is not encountered as a thing-at-hand, but as “Mitdasein”: a Dasein present to another Dasein. For Heidegger, Dasein is fundamentally, or structurally, “Mitsein,” being-with. We are existentially aware that the other is a human being, that we share this particular condition of being human; therefore, we live in a world that is, fundamentally, a human world. *Concern*, “Besorge,” determines our approach to things-at-hand; in contrast, the other human being, as a human being, calls for *solicitude*, “Fürsorge.” In the clinical situation, the patient is not only a “thing-at-hand,” but also another Dasein, and the physician is approaching the patient not only with *concern*, but also with *solicitude*. However, Heidegger states that, in our being with others, we “initially and most often” hold to “deficient modes” of solicitude: we lose ourselves in the indifference of *the ‘they’* (“das Man”), passing-one-another-by, not-mattering-to-one-another, being without-one-another. The other human being is perceived as a human being, because the very structure of Dasein is being-with—but the perception is resisted, ignored, disavowed. This is, we believe, an account of what our participants describe as the chaos, the experience of physicians not providing an anchor point.

The sense of finding an anchor point is a response to a “positive” mode of solicitude. In authentic solicitude, the fact of the patient’s humanity is not negated but acknowledged. It seems that, for the patient, this is the condition of proper care. Therefore, the patient is striving to emerge from the ‘they’, the anonymous crowd of all patients, prompting the physician to enter in a positive, rather than deficient, mode of solicitude. In order to do so, the patient must establish some sort of personal connection with the physician, as Nathalie did when she addressed the nephrologist: “Are you the one who’s stuck with me?” However, there is danger in such an attempt, because the physician might react with anger; therefore, patients are treading a fine line, walking on eggshells.

A physician who does the best for the patient is also one who understands *what* is best for the patient. Indeed, Heidegger further discusses solicitude by contrasting “leaping-in” and “leaping-ahead.” In leaping-in, one is intruding on the other’s freedom and existence as Dasein; the excessive solicitude attempts to dislodge the other’s *preoccupation*, which is the very hallmark of Dasein, of being human. Such a relationship is one of intrusion, suggestive of something like a clumsy paternalism. If Virginia’s otolaryngologist had responded, to her realistic fear of losing her eye, with words such as “Don’t worry, you will be fine”, he would have been leaping-in. In contrast, when leaping-ahead, one accompanies the other, keeping open a space of freedom for the other’s existence, who is fully recognized as another human being.^5^ Leaping-ahead requires *considerateness* (Rücksicht) and *indulgence* (Nachsicht). Too much considerateness and too little indulgence lead to leaping-in. Too little considerateness and too much indulgence lead to the indifference of the ‘they.’ When the nephrologist replies, “no, you’re the one who’s stuck with me,” he is not leaping-in, robbing Nathalie of her preoccupation; he is leaping-ahead, passing the ball to her, giving her some space and respecting her *preoccupation*. His response is not a “don’t worry, you will be fine,” it is rather a “we’re in this together.”

Here is Nathalie, trying to describe such a *considerate* and *indulgent* “leaping-ahead”:

A good physician would be someone who uses his knowledge and experience with the aim of supporting the patients. He supports them on their path. It is not a knowledge that you have innately, that you are using to show that you know things—it is to support and help the patients. A good physician for me respects his own humanity and respects his patients’ humanity. Who gets back to the basics. We’re two human beings, we’re both going to die. One has knowledge that will help me not to die.

To summarize, the patient presents to the physician both as a thing-at-hand and as a human being. In the health care environment, solicitude may be deficient. In that case, the patient is mostly a thing-at-hand. Being a thing-at-hand is dangerous, because it seems that, for our participants, it does not lead to adapted, diligent, *engaged* care. For such proper care, solicitude is needed. In other words, clinical practice embraces both the patient’s *handiness* and the patient as Dasein. However, Heidegger suggests, the danger then is one in which solicitude is leaping-in: a situation in which the physician acknowledges that the patient is another human being, but then “leaps in” and acts in the stead of the patient. When the physician is leaping-ahead, the patient is able to discern an anchor point, thereby relieved to some extent, from the burden of ascertaining the physician’s solicitude—reasonably confident that the physician will “study his case well,” as Jacques said, and do the best.

### The clinical situation

By integrating the results from the two studies, we propose that the patient experiences the physician’s *engagement-in-the-clinical-situation* as *solicitude*; this is constitutive of the anchor point. Our characterization of the physicians’ experiences finds its complement and validation in our analysis of the patients’ material. When patients are looking for manifestation of solicitude, they are ascertaining the physician’s engagement in their clinical situation. Thus, our two empirical studies, though conducted independently, provide mutually reinforcing insights on the nature of clinical practice.

Solicitude on the one hand, engagement on the other; however, the experience is not symmetrical. For the patient, the physician’s solicitude is a condition of proper, diligent, committed care—but it is uncertain. The patient may therefore deploy active strategies (not necessarily conscious) to verify, secure, and sometimes elicit the physician’s solicitude. This is not to say that the patient cannot experience solicitude for the clinician—indeed, it is very common in practice. But we would argue that the clinical situation for the *patient* is fundamentally centered on this issue of the *clinician*’s solicitude—while the converse is not true: the situation for the clinician is centered, or rather, as we will argue, gathered around the patient’s medical needs.

Our understanding of the clinical situation draws on Jean-Paul Sartre [17], who argued, following other phenomenological critiques of the classical, sharp dichotomy between subject and world, that the situation of a subject captures both the situation, “things themselves,” and the subject “myself among things”. As we argued in our previous article, “things themselves” include the materialities of the situation, but also the broader context: procedures and apparatuses, knowledge about diseases, colleagues and nurses, the family, the hour of the day and the day of the week and the season, his or her own past experiences and mentors, etc. The situation is a space of interrelations, and the patient is one among the “things themselves,” but a very particular one. In good clinical practice, the situation is pulled towards the patient, who constitutes the center of gravity of the situation. Another metaphor is the magnetic North: the physician’s solicitude orients the situation and the clinical acts towards the patient. Whereas engagement describes the practical, concrete nature of the physician’s involvement in the situation, solicitude reaches out to the patient. As the physician is gathering the situation around the patient, the patient in turn is attempting to become the focal point of the situation for the gaze of the physician. This is done by attempting to establish a human relationship, that is, by appearing to, and being recognized by the physician, as a human being, and thereby securing solicitude and engagement.

## Conclusions

Our results undermine the conceptual validity of the “dual discourse” in medicine. The dual discourse has been identified by medical anthropologists [3] and describes clinical practice as the sum of two distinct components:

Physicians must be competent; they should also embody caring qualities. “Competence” is associated with the language of the basic sciences, with “value-free” facts and knowledge, skills, techniques, and “doing” or action. “Caring” is expressed in the language of values, or relationships, attitudes, compassion, and empathy, the nontechnical or […] “personal” aspect of medicine. (p. 91)

The theoretical knowledge of the biomedical sciences and the statistical approaches of “evidence-based” medicine constitute the “scientific” component of clinical medicine, characterized by the supposedly rational, objective and value-neutral principles of technoscience. Its object is the body and the disease. But because this is considered too narrow a definition of medicine’s task, a second component is added to the first, in order to account for the patient as a human person. The dual discourse is indeed fueled by recurrent criticisms of medicine as a dehumanized and dehumanizing enterprise (see [18, 19] for classical examples; [20, 21] for recent ones; [22] for a historical perspective).

The dual discourse account of practice appears, simultaneously and paradoxically, to be self-evident and very abstract. Self-evident, because the notion of medicine as dealing with diseases is seductive; and once that is established, the necessity of a complementary notion of humane care seems obvious. Abstract, because this addition of components is not what clinical practice feels like, neither to the physicians, nor to the patients. These complementary abstractions, medicine cures diseases, and medicine cares for human beings, are reminiscent of Husserl’s epistemological critique of positivism [23]. Indeed, we believe that our phenomenological description constitutes a more faithful account of clinical practice as a unitary lived experience. The dual discourse of medicine should be decisively abandoned.

Our proposal stands in contrast to an account of clinical medicine as an applied science requiring a number of specific competencies, some technoscientific, other relational, moral, or vaguely “humane.” Engagement and solicitude are not competencies. Rather, they are proposed as an invitation to learn, through experience, the ways of engagement, and thus become a practitioner who embodies the ethos of medicine—what patients look for in the chaos they are forced to navigate, the anchor point of a physician to whom they will matter.

## Footnotes

1 We did not include in the analyses issues related to the participants’ specific experiences as patients-as-partners, except where these were related to experiences of clinical practice.

2 All interviews were conducted in French; the quotations are our direct translations in English of the verbatim transcripts. Some of the written texts may appear to have poor grammar; this is explained by the fact that we endeavored to capture the prosody of the patients’ voices. We sometimes use italics to emphasize specific moments relevant to the arguments made in the manuscript. We have also modified a number of minor details to ensure anonymity.

3 Nathalie’s words echo Arthur Frank’s notion of the “chaotic type” of illness narratives [24].

4 Issues of translation of Heidegger’s terminology are numerous and quite debatable. In this section, we mostly follow Macquarrie and Robinson [16] and translate “Besorge” by *concern* and “Fürsorge” by *solicitude*; however, we translate “Sorge” by *preoccupation* rather than *care*, to avoid any confusion with care in a more general sense, as in “health care”, or “cure versus care.”

5 We believe that the intersubjective space opened by “leaping-ahead” is close to what the psychoanalyst Donald Winnicott [25] described as a “transitional space”, but this goes beyond the scope of the present article.
